# Brown and Brite: The Fat Soldiers in the Anti-obesity Fight

**DOI:** 10.3389/fphys.2019.00038

**Published:** 2019-01-30

**Authors:** Shireesh Srivastava, Richard L. Veech

**Affiliations:** ^1^Systems Biology for Biofuels Group, International Centre for Genetic Engineering and Biotechnology (ICGEB), New Delhi, India; ^2^Laboratory of Metabolic Control, National Institute on Alcohol Abuse and Alcoholism (NIAAA), National Institutes of Health (NIH), Bethesda, MD, United States

**Keywords:** brown fat, dietary additive, exercise, metabolism, hormones

## Abstract

Brown adipose tissue (BAT) is proposed to maintain thermal homeostasis through dissipation of chemical energy as heat by the uncoupling proteins (UCPs) present in their mitochondria. The recent demonstration of the presence of BAT in humans has invigorated research in this area. The research has provided many new insights into the biology and functioning of this tissue and the biological implications of its altered activities. Another finding of interest is browning of white adipose tissue (WAT) resulting in what is known as beige/brite cells, which have increased mitochondrial proteins and UCPs. In general, it has been observed that the activation of BAT is associated with various physiological improvements such as a reduction in blood glucose levels increased resting energy expenditure and reduced weight. Given the similar physiological functions of BAT and beige/ brite cells and the higher mass of WAT compared to BAT, it is likely that increasing the brite/beige cells in WATs may also lead to greater metabolic benefits. However, development of treatments targeting brown fat or WAT browning would require not only a substantial understanding of the biology of these tissues but also the effect of altering their activity levels on whole body metabolism and physiology. In this review, we present evidence from *recent* literature on the substrates utilized by BAT, regulation of BAT activity and browning by circulating molecules. We also present dietary and pharmacological activators of brown and beige/brite adipose tissue and the effect of physical exercise on BAT activity and browning.

## Introduction

Given the widespread prevalence of obesity and associated diseases, efforts are underway to reduce the body weight gain through modulating the energy intake and/or expenditure. A large portion of the resting energy expenditure is spent on thermoregulation (Lee and Greenfield, 2015). Uncoupled respiration – a process where the oxidative phosphorylation is uncoupled from ATP generation, shivering thermogenesis, and diet-induced thermogenesis play an important part in thermal homeostasis. Overall, thermogenesis accounts for about 15% of the daily energy expenditure (van Marken Lichtenbelt and Schrauwen, 2011) or about 20% of the oxygen consumed (Rolfe and Brown, 1997). Thus, activation of uncoupled respiration could be a useful strategy to counter body weight gain. In mammals, brown adipose tissue (BAT) plays an important role in uncoupled respiration. While some previous studies had shown the existence of BAT in adult humans (Heaton, 1972; Huttunen et al., 1981; Bouillaud et al., 1983), it was the (re)demonstration of active BAT in adult humans about 10 years ago (Nedergaard et al., 2007; Cypess et al., 2009; Virtanen et al., 2009; Zingaretti et al., 2009) that has greatly increased the research efforts to understand and modulate this tissue. In humans, BAT is often seen in the cervical, upper supraclavicular area, mediastinal and perirenal regions. Due to its intense PET signal in the upper supraclavicular area (abbreviated “USA”), it became popularly known as “USA” fat (Cohade et al., 2003).

Brown adipose tissue plays an active role in thermoregulation in adult humans (Chondronikola et al., 2016). Biochemical and ultrastructural analyses identified that this tissue is rich in mitochondria and possesses a unique protein called the uncoupling protein 1 (UCP1). Other pathways, such as the glycerol-3-phosphate shuttle (Anunciado-Koza et al., 2008) and creatinine cycling (Kazak et al., 2015) may also contribute to heat generation along with UCP1 mediated uncoupling. These molecules provide the tissue with a thermogenic capacity which helps in maintaining body temperature without the need to shiver constantly. The heat thus generated is termed as non-shivering thermogenesis (NST). Some recent review articles have looked into the effect of various biochemical mediators on UCP1 activity and molecular tools used to study UCP1 functioning (Klingenberg, 2017; Ricquier, 2017). Given the potential of regulated uncoupling to dissipate energy, novel chemical uncouplers are being investigated (Ost et al., 2017) in other tissues such as WAT and skeletal muscle.

A lot of our understanding of BAT functioning comes from rodent studies. In mice, the interscapular BAT (IBAT) is the primary BAT depot, while brown adipocytes are also present in the supraclavicular region (scBAT). Several mice depots with topological similarities to human BAT-like and beige depots were identified recently (Zhang et al., 2018). Another study has shown that the gene expression pattern of mice scBAT was similar to that of human scBAT (Mo et al., 2017).

Under prolonged cold conditions, the brown fat size and activity increases, a term called BAT “recruitment.” BAT recruitment is associated with increased proliferation and differentiation of BAT precursor cells. Exposure to cold also increases BAT volume and activity in humans (Blondin et al., 2014) and also in individuals with obesity and type 2 diabetes (Hanssen et al., 2016). Increased sympathetic nervous system (SNS) activity, including on cold exposure, is a primary mechanism of BAT activation. Activated SNS releases norepinephrine which acts on the beta-adrenergic receptors on the BAT. In addition to epinephrine and norepinephrine, dopamine stimulation of brown adipocytes was also associated with increased oxygen consumption, UCP1 protein and mitochondrial mass (Kohlie et al., 2017). Prolonged exposure of mice to cold not only leads to brown fat recruitment but also to appearance of white adipocytes containing multilocular fat droplets and UCP1-expressing mitochondria – a process called “browning” of the white adipose tissue (WAT) depots. Brown-like adipocytes in WAT can arise from several origins – through the development of distinct subpopulations or through the trans-differentiation of differentiated white adipocytes (Okamatsu-Ogura et al., 2018). Additionally, the “brite” cells may also develop through the bi-directional interconversion of some cells between brite and white adipocyte phenotypes (Rosenwald et al., 2013). In spite of many molecular similarities between the BAT and brite cells, there is a differential expression of certain genes between BAT and brite cell. These include metabolic proteins (e.g., Slc27a1), inflammatory proteins (e.g., CD40 and CD137) and transcription factors (Tbx15 and Zic1) (Waldén et al., 2012; Wu et al., 2012). The gene expression profile of BAT in human infants resembles that of the classical BAT (Lidell et al., 2013), though they also express typical brite marker proteins TBX1 and CD137 (Sharp et al., 2012). The BAT in the neck of adult humans contains a mixture of classical brown and brite cells (Jespersen et al., 2013; Lidell et al., 2013). While the expression of browning genes in mice is greater in subcutaneous WAT (scWAT) compared to visceral WAT (vWAT), an opposite pattern of browning gene expression with vWAT having higher expression than scWAT was observed in humans (Zuriaga et al., 2017).

Brown adipose tissue and UCP1 levels have been shown to be involved in body weight regulation, glucose, and lipid homeostasis in mice (Kontani et al., 2005; Feldmann et al., 2009; Stanford et al., 2013). Mice strains which have a tendency to be obese have comparable BAT levels and activity but diminished browning of WAT depots (Guerra et al., 1998; Xue et al., 2007). Interestingly, transplantation of BAT from healthy mice into the visceral cavity of apoE–/– mice led to a 20% reduction in atherosclerotic lesions (Kikai et al., 2017). Similarly, transplantation of human beige tissue in mice significantly improved their metabolic parameters (Min et al., 2016). Obese humans have reduced BAT compared to those with normal weight (Orava et al., 2013) and the amount of detectable BAT correlated inversely with total, subcutaneous and visceral adiposity (Saito et al., 2009). Individuals who are BAT-positive have a reduced probability of type 2 diabetes and obesity (Cypess et al., 2009). Another study employing retrospective analysis of FDG-PET/CT scans of 4852 patients showed that BAT-positive patients had lower visceral, subcutaneous and liver fat content (Brendle et al., 2018). BAT-positive individuals have better insulin-stimulated glucose disposal compared to BAT-negative individuals (Chondronikola et al., 2014). Among patients with cardiovascular comorbidities, those with higher BAT fraction had better metabolic profiles (Franssens et al., 2017). The activated BAT was shown to be associated with reduced arterial inflammation and fatty liver (Nam and Jun, 2017). Even in newborns, those with the higher BAT at birth were shown to have lesser fat-gain over the period of 6 months (Entringer et al., 2017). Activation of BAT by cold-exposure was shown to be associated with improved glucose uptake, insulin sensitivity and reduced plasma FFA levels (Iwen et al., 2017), and lesser central adiposity (Green et al., 2017). Activated BAT is also associated with other physiological benefits such as amelioration of obesity-associated reduction in male fertility (Liu H. et al., 2017) and improved menstrual regularity in rat model of Polycystic ovary syndrome (PCOS) (Yuan et al., 2016). Thus, BAT recruitment has been suggested as an anti-obesity agent in humans (Yoneshiro et al., 2013). Individuals with active BAT weighed on an average 4 kg less than BAT negative individuals (Lee et al., 2010). Leaner individuals had about 50% higher UCP1 mRNA levels. Interestingly, the UCP1 levels accounted for about 50% of BMI variance (Lee et al., 2011).

Given the strong correlation between active BAT and positive metabolic and physiological responses, there is an intense interest in understanding the biology and regulation of this tissue. The level of interest can be gleaned from the fact that the search for the term “brown adipose tissue” in Pubmed produced a list of over 10000 articles, most of which were published in the past decade. While there are many reviews that cover specific aspects of brown and brite adipocyte biology and some of the recent ones are referenced in this review, our aim is to provide an integrative overview of the recent literature and current understanding of brown/brite adipocyte biology and functioning.

## Substrates Utilized by Brown Fat for Heat Production

Brown fat is a metabolically active tissue that can metabolize a variety of substrates for the production of heat. A major source for short-term activity is the intracellular lipid that is stored in the form of multi-locular droplets. However, knockout of essential enzymes of lipolysis (Schreiber et al., 2017; Shin et al., 2017) indicate that lipolysis is not essential for cold-induced thermogenesis in mice. However, inhibition of intracellular lipolysis by feeding nicotinic acid was associated with a diminished increase in cold-induced BAT activity in men (Blondin et al., 2017a).

Brown fat can also metabolize a variety of extracellular substrates, primary among which is glucose. The rapid uptake of glucose by this tissue is the reason for its prominent display during the FDG-PET imaging. It has been suggested that the glucose can be used for *de novo* fatty acid synthesis which is then channeled to a pool of triacylglycerol (TAG) which is rapidly hydrolyzed to yield fatty acids that can serve as substrates for increased thermogenesis (Irshad et al., 2017). Circulating lipids and lipoproteins are also utilized by BAT (Bartelt et al., 2011; Berbée et al., 2015; Khedoe et al., 2015; Blondin et al., 2017b). The clearance of glucose and triglycerides by cold-activated BAT can account for about two-thirds of total increase in substrate clearance (Bartelt et al., 2011). Circulating acylcarnitines (Simcox et al., 2017), as well as lipoproteins (Hoeke et al., 2016) can also be utilized by activated BAT as substrates. Lipid oxidation was shown to be important for BAT thermogenic function (Gonzalez-Hurtado et al., 2018). A correlation between BAT activity and serum HDL cholesterol levels has been reported (Bartelt et al., 2017). Thus, the BAT, when activated, could be an important organ for clearance of glucose and lipid species.

## Circulating Modulators of Brown Adipose Activity and Browning

**FIGURE 1:**
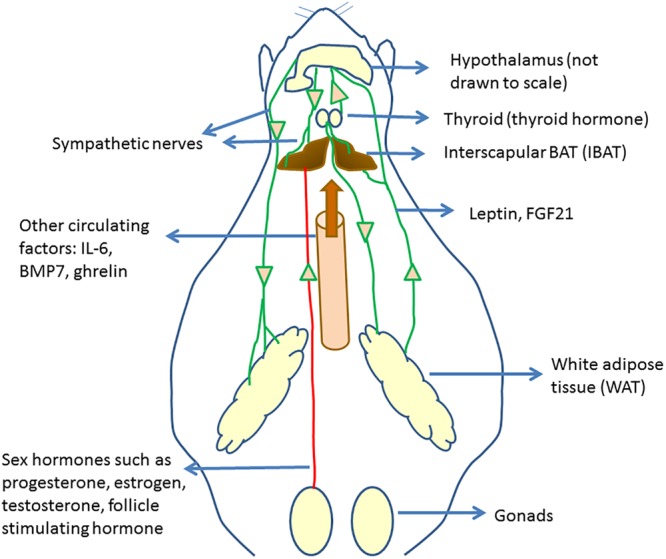
Circulating regulators of BAT and their origin. Hypothalamus plays an important role in regulating brown fat activity through regulating the sympathetic nervous system activity. Several circulating regulators impact BAT functioning and browning. Many of these act through increasing the sympathetic nervous activity to BAT and WAT, increasing UCP1 expression.

Several previous and recent studies have shown that BAT activation and WAT browning are regulated by the actions of various hormones. The regulation of BAT activity and browning by hormonal mediators was the subject of some recent review articles (Hu and Christian, 2017; Rodríguez et al., 2017; Ludwig et al., 2018). In this section, we present some of the recent literature reports on BAT regulation, linking those to the overall understanding of BAT physiology and function. Figure 1 provides an overall picture of the regulation of brown fat activity and browning by hormones and circulating factors.

Hyperinsulinemia, induced by daily injection of insulin, was associated with a reduction in IWAT and IBAT respiratory activity (Dallon et al., 2018). Leptin, a hormone released by WAT and known for its appetite suppressing effects, was shown to increase SNS activity to BAT (Enriori et al., 2011). However, the IBAT SNS activity was shown to be not necessary for leptin-induced weight loss (Côté et al., 2018). Proopiomelanocortin (POMC) and Agouti-related protein (AgRP) neurons play a role in leptin-mediated increased SNS activity to BAT, while the AgRP are the major regulators of increased SNS activity to the inguinal fat in response to leptin in mice (Bell et al., 2018). Significantly reduced levels of the β3-adrenergic receptor, PGC-1α, and UCP1, were found in the leptin-deficient ob/ob (–/–) mice (Martins et al., 2017) which were improved by injection of capsules containing poly-L-lysine-embedded engineered 3T3-L1 adipocytes constitutively expressing leptin (DiSilvestro et al., 2016).

Increased thyroid hormone activity is known to promote energy expenditure. One study showed that both hypothyroidism, as well as hyperthyroidism, led to increased WAT browning in mice (Weiner et al., 2016). The study showed that hyperthyroid mice had higher BAT mass and activity than the hypothyroid mice. The thyroid hormone T3 can increase SNS activity to BAT, increasing BAT activity (López et al., 2010). Treatment with T4 or administration of T3 to VMH was shown to be associated with the browning of WAT (Martínez-Sánchez et al., 2017a). In humans too, the cold-induced increase in energy expenditure was shown to be associated with circulating T3 levels (Gavrila et al., 2017). The action of T3 on BAT activity was shown to be mediated by a reduction of endoplasmic reticulum stress in the VMH (Martínez-Sánchez et al., 2017b). Angiotensin type 2 receptor (AT2R) was shown to play a role in T3-induced upregulation of browning genes in WAT (Than et al., 2017) and an AT2R agonist was shown to increase WAT browning. On the other hand, deleting the AT1aR was shown to be associated with IWAT browning (Tsukuda et al., 2016). Injection of angiotensin 1–7 peptide through a micro-osmotic pump was shown to increase in BAT size and UCP1 levels as well as increase thermogenesis in subcutaneous WAT without affecting UCP1 levels there (Morimoto et al., 2018). A thyroid receptor beta (TR-β) specific agonist GC-1 was shown to increase energy expenditure and prevent weight gain in rats (Villicev et al., 2007). The molecular mechanisms involved in the TH-induced increase in thermogenesis was reviewed in Weiner et al. (2017) and involves both direct activation of thyroid hormone receptors in the adipose tissues as well as indirectly through the activation of hypothalamic neurons. A recent study has identified the carbohydrate response element binding protein (ChREBP) as one of the targets of T3 which regulates the UCP1 expression in brown adipocytes (Katz et al., 2018).

Sex hormones play an important role in regulating brown fat function. As several studies have shown that females have higher BAT activity than males, it is likely that estrogen levels impact BAT activity (Frank et al., 2018). Reduced levels of estradiol (E2), an ovary-derived hormone, are associated with reduced BAT activity (López and Tena-Sempere, 2016) and E2 treatment can increase BAT activity by activating hypothalamic AMPK (López and Tena-Sempere, 2017). The roles of E2 in regulating thermogenesis were recently reviewed (González-García et al., 2017). Pharmacological activation of estrogen receptor β(ER-β) was shown to increase BAT volume and energy expenditure (Ponnusamy et al., 2017). Progesterone, a hormone associated with gestation, was shown to cause a brown-to-white conversion of BAT in mice (McIlvride et al., 2017). Removal of BAT prior to conception led to maternal and fetal hyperlipidemia and larger fetuses. Inhibiting follicle stimulating hormone (FSH) through a polyclonal antibody was shown to induce beiging, activate BAT and thermogenesis and reduce WAT (Liu P. et al., 2017). BAT was shown to have the highest expression of Follistatin (Fst), previously known as FSH-suppressing protein. Overexpression of Fst was shown to increase BAT mass as well of browning of WAT (Singh et al., 2017). These results suggest that FSH has an inhibitory effect on BAT activity and browning. Castration was shown to increase browning of IWAT in mice (Hashimoto et al., 2016).

Some other hormones or peptides have been reported to affect BAT functioning. These include ghrelin, which negatively correlated with BAT activity (Chondronikola et al., 2017) and erythropoietin (EPO), which was shown to promote thermogenic activity (Kodo et al., 2017).

Small molecule circulating regulators of BAT physiology include hydrogen sulfide (H_2_S) (Soriano et al., 2018), pyruvate (Soto et al., 2018) and abscisic acid (Sturla et al., 2017).

Brown fat is also a source of various BATokines which can impact other organs. FGF21 is a well-studied batokine, though it can also be released by liver and WAT. FGF21 can act on BAT and WAT through the central nervous system and increase the SNS activity, leading to weight loss and increased energy expenditure (Bookout et al., 2013; Owen et al., 2014; Douris et al., 2015). FGF21 can also act in an autocrine manner on the tissue. FGF21 can also lead to browning of WAT through increased PPAR-γ (Dutchak et al., 2012) and PGC1-α activity (Fisher et al., 2012). A clinical trial showed a reduction in body weight and improvements in lipid metabolism in obese patients on treatment with LY2405319, analog of FGF-21 (Gaich et al., 2013). Batokines and their effects have been reviewed in Villarroya et al. (2017).

## Dietary and Plant-Derived Molecules

**FIGURE 2:**
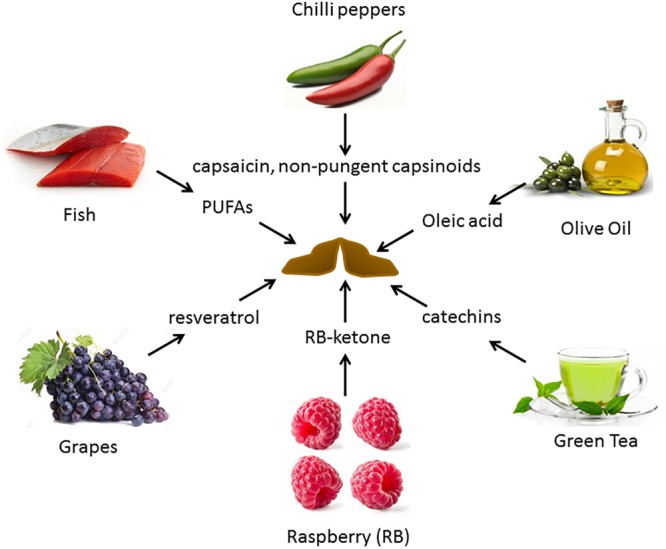
Food-derived molecules shown to be involved in BAT activation and browning, and their sources. Several diet-derived molecules have been shown to activate brown fat and browning. These are obtained from both plant and animal products.

Alterations in diets and major dietary components have been shown to be associated with variation in the activity of BAT. Figure 2 depicts some of the food items whose derived compounds have been shown to increase BAT activity and browning of WAT.

### Macromolecular Composition of Diets

The macromolecular composition of diets can affect BAT activity and browning. A comparison of several diets showed that a high protein diet was associated with higher amounts of BAT (de Macêdo et al., 2017). However, other studies have shown that diets low in protein diet were associated with increased BAT activity and browning (Pereira et al., 2017; Kitada et al., 2018). Some studies have observed that the source of protein affects diet-induced thermogenesis (DIT) and BAT UCP1 levels (Ezoe et al., 2016; Li T. et al., 2018). The BAT response on a high fat diet was time-dependent – shorter (2–4 week) high-fat diet increase UCP1 levels in BAT, while longer feeding with HFD (20 weeks) bringing them back to normal levels (Ohtomo et al., 2017).

Surprisingly, sucrose intake was shown to increase BAT activity (Maekawa et al., 2017; Velickovic et al., 2018). Addition of dietary fiber to a high-fat diet (HFD) increased lipolysis and the levels of various thermogenic proteins in WAT (Han S.-F. et al., 2017).

### Dietary Components Known to Increase BAT Activity

Long-term feeding of specific diets may cause alterations in the BAT composition and activity. Our work has shown that a high fat, low carbohydrate ketogenic (KG) diet (Srivastava et al., 2013) increased BAT volume and the expression of mitochondrial proteins in BAT in mice. A novel dietary additive, which can be given along with a normal carbohydrate-level diet and yet can increase blood ketone levels, was developed in the lab of R.L. Veech. This ketone ester, when given to mice in liquid diets, produced blood ketone (β-hydroxybutyrate) levels much greater than that achieved with the KG diets without the need to restrict carbohydrate intake (Srivastava et al., 2012). The increased ketone levels were associated with several positive metabolic effects. These include a significant activation of BAT and mitochondrial biogenesis in this tissue, increased UCP1 levels in WAT. Dietary administration of medium chain triglycerides (MCTs) (Zhang et al., 2015) as well as MCT-enriched diacylglycerol oil (MCE-DAG) (Kim et al., 2017), which also increase blood ketone levels, was shown to activate BAT. Azelaic acid, a pharmacological activator of Olfr544, was shown to induce ketogenesis in the liver and increased UCP1 expression in the BAT (Wu et al., 2017). All these results indicate that increased blood ketone levels can modulate brown fat activity. Other diet-derived small molecule BAT activators have also been identified. These include butyrate (Li Z. et al., 2018), acetate (Sahuri-Arisoylu et al., 2016) and succinate (Mills et al., 2018). These studies suggest that elevation of TCA cycle metabolites can trigger changes in BAT activity. Table 1 lists some of the diet-derived small molecules shown to increase BAT activity and browning of WAT.

**Table 1:** Some diet-derived small molecules known to activate brown fat and/or browning.

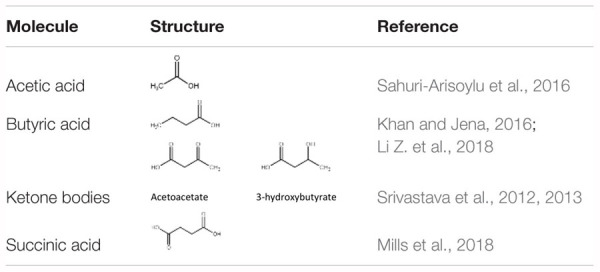

There is a significant interest in identifying dietary additives that can improve BAT activity. Several dietary BAT modulators have been identified. Figure 2 provides a summary of reported dietary molecules that increase BAT activity and browning.

Feeding mice a diet rich in fish oil (Bargut et al., 2016), olive oil (Shin and Ajuwon, 2018) or PUFAs (Crescenzo et al., 2017; Pahlavani et al., 2017; Ghandour et al., 2018) or conjugated linoleic acid (Shen and McIntosh, 2016) was shown to activate BAT and browning in rodents.

Chronic cold exposure was associated with increased bile acid production by the liver and modulation of the bile acid production modulated the thermogenic response (Worthmann et al., 2017). Studies have shown activating effect of bile acids on BAT activity in rodents (Watanabe et al., 2006; Eggink et al., 2018) and humans (Broeders et al., 2015).

Supplementation with non-pungent capsinoids (compounds associated with the hotness of peppers) (Yoneshiro et al., 2012) as well as grains of paradise (Sugita et al., 2013) was associated with increased thermogenesis. Activation of the transient receptor potential cation channel subfamily V member 1 (TRPV1) by these compounds (Baskaran et al., 2016) leads to increased SNS activity to induce BAT (Saito and Yoneshiro, 2013). Selective ablation of inguinal WAT (IWAT) sensory neurons by capsaicin treatment was shown to reduce the norepinephrine turnover not only in the IWAT but also in the IBAT, and reduce UCP1 expression in IBAT (Nguyen et al., 2018). Similarly, loss of TRPV2 was associated with impaired thermogenic response to β-adrenergic stimulation and poor cold tolerance (Sun et al., 2016a, 2). Expression of TRPV2 is significantly increased during brown fat differentiation, and TRPV2-agonists inhibited the brown adipocyte differentiation (Sun et al., 2016b). Human studies have shown that increased energy expenditure following capsaicin treatment was observed only in individuals with the active BAT (Sun et al., 2018). Supplementation of vanillic acid also increases BAT activity (Jung et al., 2018). These studies have shown that BAT plays a role in the increased energy expenditure following intake of “hot” foods and have elucidated the underlying mechanisms, providing new targets to modulate BAT activity.

Several studies have shown that intake of tea-derived compounds is associated with increased thermogenic capacity in mice (Neyrinck et al., 2017) and humans (Nirengi et al., 2016; Yoneshiro et al., 2017).

Resveratrol (RSV), a flavonoid found on the skin of grapes, and related compounds have been shown to activate BAT and increase thermogenesis (Andrade et al., 2014; Ku et al., 2016; Li et al., 2017) likely through AMPKα activation (Wang et al., 2017). A combination of RSV with Quercetin, active compound from onion peels (Arias et al., 2017), and pentamethyl quercetin (PMQ) (Han Y. et al., 2017) was shown to induce BAT activation and browning.

Dietary supplementation of raspberry to mice on a high-fat diet (Xing et al., 2018) or oral treatment with raspberry ketone (Leu et al., 2018) was recently shown to increase beiging through AMPK activation (Zou et al., 2018).

While the list given in this section is not exhaustive, it is clear that BAT and WAT are responsive to diet composition, type of lipid and to several natural compounds. This also calls for an appropriate control diet as well as a careful comparison of the macro and micronutrient composition of diets while designing dietary studies involving BAT activators.

## Pharmacological Activators of Brown and Beige Fat

Several pharmacological activators of brown fat and browning have been reported. These include β3-adrenergic receptor agonist (Cypess et al., 2015), PPAR-γ activators (Xie et al., 2017; Loh et al., 2018; Merlin et al., 2018), PGC-1α stabilizers (Pettersson-Klein et al., 2018), PPAR-α agonist (Rachid et al., 2018), AMPK activators (Kim et al., 2016, 2018; Tokubuchi et al., 2017) and PDE5 inhibitors Sildenafil and Tadalafil (Maneschi et al., 2016; Li S. et al., 2018). Several other pharmacological modulators of BAT activity and browning have been reported. While some of them are expected from our understanding of brown fat biology, the other modulators identified could be used to gain further insights into the mechanisms of regulation of brown fat activation and browning.

## Effect of Physical Exercise on BAT and Browning

Physical exercise can have beneficial effects on general metabolism and physiology. Several rodent studies showed increased BAT activity and browning of WAT in rodents on exercise (De Matteis et al., 2013; Aldiss et al., 2017; Peppler et al., 2018). However, a systematic review concluded that regular exercise is not a major stimulus for increased BAT activity even though an increase may be observed in animals consuming high-fat diets or with low endogenous UCP1 levels (Flouris et al., 2017). Most human studies have shown a negative correlation of BAT activity with exercise (Motiani et al., 2017; Trexler et al., 2017). Endurance-trained athletes have reduced BAT activity compared to sedentary controls (Trexler et al., 2017). Endurance training was also not associated with beiging of abdominal or subcutaneous WAT (Tsiloulis et al., 2018). Two weeks of exercise training decreased insulin-mediated glucose uptake by BAT in healthy middle-aged men (Motiani et al., 2017). Thus, human studies have generally shown that metabolic benefits of exercise are not mediated by increased BAT activity and browning.

Several exercise-induced mediators have been suggested to play a role in browning of WAT in rodents. These include myokines such as Irisin, a hormone released by skeletal muscles whose levels increase following exercise (Boström et al., 2012), IL-6 (Knudsen et al., 2014) and β-amino isobutyric acid (BAIBA) (Roberts et al., 2014), etc. While the initial findings of existence of irisin were questioned due to non-specificity of the antibodies (Atherton and Phillips, 2013; Albrecht et al., 2015), a mass spectrometry-based analysis confirmed the presence of irisin in human plasmas and its increase following exercise (Jedrychowski et al., 2015). In humans, habitual physical activity was shown to be positively correlated with serum irisin levels (Buscemi et al., 2018) but a lower active BAT (Singhal et al., 2016). A systematic review of the literature has concluded that it may not be possible to conclude an association between physical activity and Irisin or PGC-1α because of lack of precision of available methods (Dinas et al., 2017). Thus, further research is needed to evaluate the effects of myokines on brown fat physiology and browning process, taking into consideration that trained individual may show a divergent response than untrained/sedentary individuals (Vosselman et al., 2015).

## Conclusion and Future Directions

It is now clear that BAT is a metabolically active tissue and when activated, can clear very high levels of glucose and lipids on a per weight tissue basis. Increased BAT activity is associated with several metabolic benefits such as reduced body weight and improved glucose control. The beiging of WAT holds promise to further increase the metabolic benefits.

An important, less-emphasized factor regulating the activity of this tissue is the ambient temperature. Most of our knowledge has come from studies conducted under cold conditions which lead to chronic BAT activation. Housing at or near thermoneutral temperature is associated with significantly reduced basal metabolic rate and UCP1 levels and higher body fat (Shi et al., 2017). Chronic exposure to thermoneutral and warm conditions significantly reduce the levels and activity of this tissue in rodents (Cui et al., 2016) and humans (Turner et al., 2016). Warmer temperatures are also associated with higher incidences of diabetes and glucose intolerance in humans (Blauw et al., 2017). As most humans live at or near thermoneutrality, only a small fraction of the total BAT may be active in humans under normal living conditions. Similarly, ambient temperature may also affect the response to “inducers” of BAT activity. For BAT research to be applicable to humans, a careful choice of ambient temperature is needed.

While physical exercises of different modalities are generally associated with improved metabolic parameters, studies generally suggest a divergent response of WAT browning to exercise in rodents vs. humans. Human studies have generally shown a reduced BAT activity and no significant effect on browning, while rodent studies have generally shown browning of WAT by exercising. It is plausible that this divergent response may be related to the different ambient temperatures, along with other factors such as greater genetic and dietary diversity of humans compared to the mice studied. Nonetheless, the tantalizing possibility exists of further improving the benefits of exercise in humans through a parallel activation of browning by environmental, dietary or pharmacological treatments. Various exercise-induced factors have also been identified, with the goal to extend the metabolic benefits of exercising to sedentary individuals.

Given our increased understanding of the metabolic effects of the activated BAT, novel approaches to increase its activity are being explored. Using optical and electrical genetic stimulation of specific neurons in the sympathetic nervous offers an interesting option (François et al., 2017) to modulate the activity of BAT or to induce browning in specific depots. Oral genetic therapy to manipulate BAT has been reported in mice (Huang et al., 2016) and was able to increase BAT mass and activity. Similarly, stem cells derived from rat and human WAT were successfully differentiated into a three dimensional BAT using hydrogels (Yang et al., 2017). The effect of gut microbiota on BAT activity and browning is increasingly being recognized (Moreno-Navarrete et al., 2018) and would likely be a fertile area of research.

Novel small molecule activators of BAT and brite cells are being developed, along with novel delivery systems such as functionalized nanoparticles (Xue et al., 2016) and lipid nanocarriers (Zu et al., 2017). Such delivery systems hold promise for precise and efficient delivery of bioactives to their target site(s). Similarly, several dietary additives that activate BAT and browning have been reported. However, long-term studies with pharmacological and dietary treatments are needed. Future studies can also investigate the combined dietary and pharmacological treatments. Further research is likely to generate more information on BAT and brite biology and its interaction with various other physiological processes.

## Author Contributions

SS and RV wrote the manuscript.

## Conflict of Interest Statement

The authors declare that the research was conducted in the absence of any commercial or financial relationships that could be construed as a potential conflict of interest.
